# Use of Probiotics to Control Aflatoxin Production in Peanut Grains

**DOI:** 10.1155/2015/959138

**Published:** 2015-06-28

**Authors:** Juliana Fonseca Moreira da Silva, Joenes Mucci Peluzio, Guilherme Prado, Jovita Eugênia Gazzinelli Cruz Madeira, Marize Oliveira Silva, Paula Benevides de Morais, Carlos Augusto Rosa, Raphael Sanzio Pimenta, Jacques Robert Nicoli

**Affiliations:** ^1^Departamento de Microbiologia, Instituto de Ciências Biológicas, Universidade Federal de Minas Gerais, Bloco J, 4º Andar, Sala 171, Avenida Antônio Carlos 6627, CP 486, Pampulha, 31270-901 Belo Horizonte, MG, Brazil; ^2^Laboratório de Microbiologia Ambiental e Biotecnologia, Universidade Federal do Tocantins, ALC No. 14 Campus Universitário, Avenida NS 15 Bloco II Sala 05, 77.001-090 Palmas, TO, Brazil; ^3^Fundação Ezequiel Dias, Laboratório de Micologia e Micotoxinas, Rua Conde Pereira Carneiro 80, 30510010 Belo Horizonte, MG, Brazil

## Abstract

Probiotic microorganisms (*Saccharomyces cerevisiae* var. *boulardii*, *S. cerevisiae* UFMG 905, and *Lactobacillus delbrueckii* UFV H2b20) were evaluated as biological control agents to reduce aflatoxin and spore production by *Aspergillus parasiticus* IMI 242695 in peanut. Suspensions containing the probiotics alone or in combinations were tested by sprinkling on the grains followed by incubation for seven days at 25°C. All probiotic microorganisms, in live and inactivated forms, significantly reduced *A. parasiticus* sporulation, but the best results were obtained with live cells. The presence of probiotics also altered the color of *A. parasiticus* colonies but not the spore morphology. Reduction in aflatoxin production of 72.8 and 65.8% was observed for *S. boulardii* and *S. cerevisiae*, respectively, when inoculated alone. When inoculated in pairs, all probiotic combinations reduced significantly aflatoxin production, and the best reduction was obtained with *S. boulardii* plus *L. delbrueckii* (96.1%) followed by *S. boulardii* plus *S. cerevisiae* and *L. delbrueckii* plus *S. cerevisiae* (71.1 and 66.7%, resp.). All probiotics remained viable in high numbers on the grains even after 300 days. The results of the present study suggest a different use of probiotics as an alternative treatment to prevent aflatoxin production in peanut grains.

## 1. Introduction

Peanut (*Arachis hypogaea*) is one of the major food crops cultivated throughout the tropics and subtropical regions and its annual world production is of approximately thirty-eight million tons [1]. However, this grain is very susceptible to mycotoxin contamination, especially by aflatoxins. Aflatoxins are secondary metabolites produced by three filamentous fungal species:* Aspergillus flavus*,* A. parasiticus*, and* A. nomius* [2, 3]. The aflatoxins can be classified as B_1_, B_2_, G_1_, and G_2_ according to their fluorescence under ultraviolet light and molecular weight and strains of* A. parasiticus* can produce all of them [4]. The B_1_ aflatoxin is recognized by the International Agency for Research on Cancer as a group 1 carcinogenic substance, whereas aflatoxins B_2_, G_1_, and G_2_ are classified as possible carcinogenic substances [5].

Aflatoxin contamination in peanuts is a great economical and public health concern [6] and many studies search for efficient methodologies capable of controlling filamentous fungal growth in pre- and postharvest. Use of GRAS (Generally Regarded as Safe) substances and antagonistic microorganisms has been tested with some success [7–9]. Antagonism is one of the most important phenomena observed in microecological relationships, and the principal mechanism responsible for the beneficial effect of probiotic yeasts and bacteria. Probiotics are defined by the Food and Agriculture Organization/World Health Organization [10] as “live microorganisms which when administered in adequate amounts confer a health benefit on the host.” An interesting possibility of using the antagonistic ability of probiotics would be to impair growth and/or aflatoxin production by phytopathogenic fungi during storage of grains.

Shetty and Jespersen [11] reported that* Saccharomyces cerevisiae* and lactic acid bacteria (LAB) can reduce the toxic effects of mycotoxins in foods by absorption on their cell walls. The LAB are known as predominant participants in many industrial food processes, especially in vegetables, meats, and dairy fermentations, where they can prevent contamination by harmful microorganisms.* Saccharomyces cerevisiae* is the most important microorganism responsible for production of alcoholic beverages and biofuel. Both LAB and* Saccharomyces* (particularly* S. cerevisiae* var.* boulardii*) have already been described for their use as probiotics [12–14].

*S. cerevisiae* (UFMG 905) reduced the translocation levels of pathogens, promoted the host immunomodulation, decreased the mortality and helped in the preservation of liver tissue and gut barrier integrity, and reached population levels potentially functional in the gastrointestinal tract in mice [15–17].* Lactobacillus delbrueckii* (H2b20) protected germfree mice against infection with pathogens and stimulated the cytokines production. Due to this, these microorganisms were elected to be tested as food protectors in this study [18].

The objective of this work was to evaluate the capabilities of three probiotic microorganisms (two strains of* S. cerevisiae* and one of* L. delbrueckii*) to reduce the sporulation and aflatoxin production by* A. parasiticus* in peanuts.

## 2. Materials and Methods

### 2.1. Peanut Grains

Autoclaved peanuts grains, cultivar IAC Caiapó, available from Instituto Agronômico de Campinas were used. The grains had medium size, brownish color, high oil content, and no aflatoxin presence and were produced in the 2009/2010 crop.

### 2.2. Microorganisms

*Aspergillus parasiticus* IMI 242695 isolated from contaminated food products and aflatoxin producer (B_1_, B_2_, G_1_, and G_2_) was obtained from the International Mycological Institute (UK).* Saccharomyces boulardii* 17 was obtained from MERCK/SA (Rio de Janeiro, RJ, Brazil),* S. cerevisiae* UFMG 905 pertained to the Collection of Microorganisms and Cells of Federal University of Minas Gerais (UFMG), and* Lactobacillus delbrueckii* H2b20 was isolated from a healthy newborn and pertained to the culture collection of the Laboratório de Ecologia e Fisiologia de Microorganismos (Departamento de Microbiologia, Universidade Federal de Minas Gerais, Belo Horizonte, MG, Brazil).

### 2.3. Sample Preparation: Growth, Resuspension, and Dilution

#### 2.3.1. *Aspergillus parasiticus* IMI 242695

Spores were obtained as described by Prado et al. [19]. Spore concentration was determined using a hemocytometer and adjusted to 1 × 10^6^ spores/mL in 50 mL of 0.1% Tween 80.

#### 2.3.2. Yeasts

*Saccharomyces boulardii* and* S. cerevisiae* UFMG 905 cells were obtained by culture on YM agar medium (2% glucose, 0.5% peptone, 0.3% malt extract, 0.3% yeast extract, and 2% agar) incubated for 24 h at 37°C. Colonies were collected and suspended in 0.1% Tween 80 and the cell concentration was determined using a hemocytometer and the suspension was adjusted to 1 × 10^8^ cells/mL in a final volume of 50 mL.

#### 2.3.3. *Lactobacillus delbrueckii*

Bacterial cells were obtained by culture on de Man, Rogosa and Sharpe (MRS) agar medium incubated for 24 h at 37°C in aerobic static conditions. Colonies were collected and suspended in 0.1% Tween 80 and the cell concentration was determined using a hemocytometer and the suspension was adjusted to 1 × 10^8^ cells/mL in a final volume of 50 mL.

### 2.4. Biological Control Assays

Autoclaved peanuts grains were used in the following eleven experimental groups (15 g in each): (1) first positive control: grains inoculated with 2.5 mL of* A. parasiticus* spore suspension and 2.5 mL of 0.1% Tween 80; (2, 3 and 4) negative controls: grains inoculated with 2.5 mL of each yeast or bacterial suspension plus 2.5 mL of 0.1% Tween 80; (5, 6 and 7) simple antagonistic tests: grains inoculated with 2.5 mL of each yeast or bacterial suspension plus 2.5 mL of* A. parasiticus* spore suspension; (8) second positive control: grains inoculated with 2.5 mL of* A. parasiticus* plus 5 mL of 0.1% Tween 80; (9, 10, and 11) combined antagonistic tests: grains inoculated with 2.5 mL of combined yeast or bacterial suspension in a factorial association for inoculation with two microorganisms (*L. delbrueckii* +* S. cerevisiae*;* S. boulardii* +* S. cerevisiae*; and* S. boulardii* +* L. delbrueckii*) plus 2.5 mL of* A. parasiticus* spore suspension. All the inoculations were performed by sprinkling of grains with microbial suspensions followed by incubation for seven days at 30°C.

### 2.5. Aflatoxin Quantification

The aflatoxin quantification was performed by fluorodensitometry at 365 nm using a fluorodensitometer (model CS9301, Shimadzu Corp., Kyoto, Japan). Concentration of aflatoxin B_1_ was calculated by comparing the fluorescent intensity of sample spots with known standard amounts spotted on the same plate in the range from 0.8 to 0.88 ng [20].

### 2.6. Microbial Cell Viability on Peanut Grains

To determine the evolution of microbial cell viability on peanuts during storage, 15 g of peanut grains was placed in an plastic film covered Erlenmeyer flask (250 mL) and soaked with 2.5 mL of each yeast or bacterial suspension and the mixture was incubated at 25°C. At regular time intervals (weekly until 21 days and monthly until 330 days), viable cell counts were performed as follows. Three inoculated grains were introduced in three assay tubes containing 10 mL of sterile saline. The tubes were agitated for approximately 30 s, and an aliquot of the supernatant was submitted to serial decimal dilution up to 10^−5^ in saline sterile solution. Then, 50 *μ*L of each dilution was plated onto MRS or YM agar media for* Lactobacillus* and yeasts counts, respectively, and incubated at 30°C for 48 h. After incubation, viable cell number was determined and the results expressed as decimal logarithm of colony forming units per grain (log_10_ CFU/grain). Culture inoculation and counting were automatically performed by spiral plate and colonies counter (IUL Instruments, Barcelona, Spain).

### 2.7. *In Vitro* Antagonism Assay

To evaluate a possible* in vitro* antagonism of yeast and* Lactobacillus* probiotics against* A. parasiticus*, the agar double layer diffusion test was described by Tagg et al. [21] with some modifications. The antagonistic effect was evaluated using live and inactivated cells of each probiotic. In the assay using live cells (Assay 1) Petri dishes containing adequate medium (agar MRS or YM for bacteria or yeasts, resp.) were inoculated with 10 *μ*L of yeast or bacterial suspension in the center and incubated at 37°C during 24 h. After incubation, 3.5 mL of MEA medium (1.2% malt extract, 2% agar) supplemented with 10 *μ*L of* A. parasiticus* spore suspension was spread onto the agar surface and the dishes were incubated at 25°C for seven days. In the assay using inactivated cells (Assay 2), after inoculation on the center of the Petri dish, the yeast or bacterial spot was killed by chloroform vapor exposition for 20 min. Then, Petri dishes were held open in a laminar flow hood until complete removal of residual chloroform and the dishes were treated as in Assay 1. After incubation, presence of growth inhibition zone and alterations in appearance of the phytopathogen were observed.

### 2.8. Determination of Number and Morphology of* A. parasiticus* Spores

This determination was performed with the same Petri dishes used for the* in vitro* antagonism assay described above. The spore count was done as follows: 10 mL of 0.1% Tween 80 and ten glass beads were added to each plate, and after shaking, the supernatant was transferred to a conical tube (50 mL). After homogenization, spore concentration in the supernatant was determined using a hemocytometer. The results were expressed as spores mL. To evaluate morphological alterations, 20 *μ*L from each suspension was stained with cotton blue and visualized using an optical microscopy (Leica DM 750), and the images were captured and analyzed with the Leica microsystems DFC 425 software.

### 2.9. Statistical Analysis

All the experiments followed a randomized design with five replicates each. Data were submitted to Kolmogorov-Smirnov test of normality and then compared by variance analysis and Tukey test at 5% of significance. Statistical analysis was performed using the Sisvar 5.3 software (UFLA, Lavras, MG, Brazil).

## 3. Results and Discussion

*Saccharomyces boulardii*,* S. cerevisiae* UFMG 905, and* L. delbrueckii* were able to reduce significantly* A. parasiticus* sporulation as shown in Table 1. These reductions were observed with the utilization of both viable and inactivated yeasts and bacteria, but a higher reduction was obtained with live cells.

**Figure 1:**
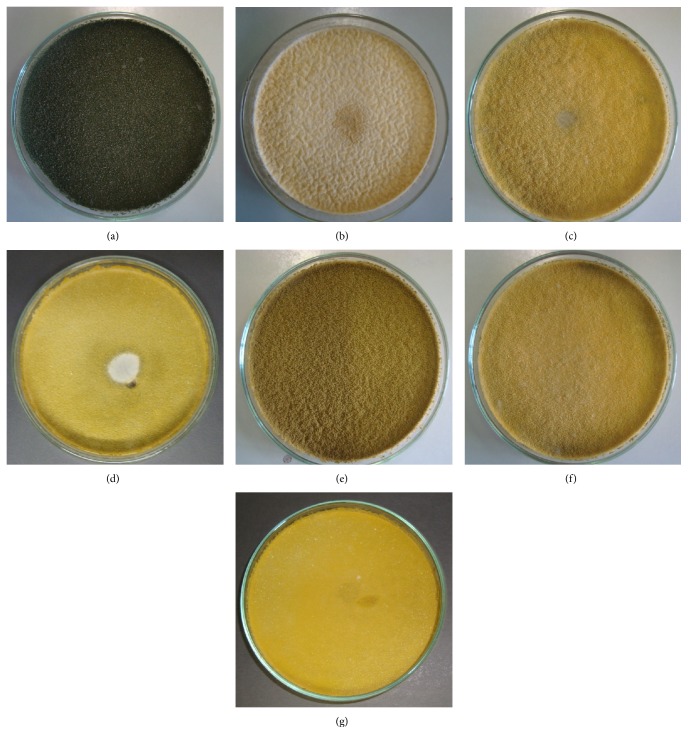
Positive control:* A. parasiticus* IMI 242695 (10 *μ*L at 10^6^ spores/mL) incubated for seven days at 30°C in semisolid malt agar (a). Group 1, live antagonistic cells (10 *μ*L 10^8^ cells/mL) incubated for seven days at 30°C.* L. delbrueckii* × overlapping culture (10 *μ*L 10^6^ spores/mL of* A. parasiticus* IMI 242695) (b);* S. boulardii* × overlapping culture (c);* S. cerevisiae* strain UFMG 905 × overlapping culture (d). Group 2, antagonistic cells inactivated by chloroform vapors (10 *μ*L a 10^8^ cells/mL) incubated for seven days at 30°C.* L. delbrueckii* × overlapping culture (10 *μ*L 10^6^ spores/mL of* A. parasiticus* IMI 242695) (e);* S. boulardii* × overlapping culture (f);* S. cerevisiae* strain UFMG 905 × overlapping culture (g).

Additionally, in these* in vitro* experiments with the use of viable and inactivated microorganisms, a color alteration in* A. parasiticus* appearance was observed when compared to the control group (olive green to yellow) (Figures 1(a)–1(g)). This alteration, due to the reduction in spore production, represents a considerable vantage for the use of the tested probiotics since this effect can reduce the pathogen dispersion in stored grains. On the other hand, there was no inhibition of the mycelium growth as well as no structural modifications of* A. parasiticus* spores which remained globose, rough (with spines), and hyaline.

In relation to the ability to reduce spore production, there was no statistical significant difference among the three viable microorganisms, despite the tendency to a better result for the* Lactobacillus*. Similar results were observed by Onilude et al. [22] using six lactic acid bacteria (LAB) (*L. fermentum* OYB,* L. fermentum* RS2,* L. plantarum* MW,* L. plantarum* YO,* L. brevis* WS3, and* Lactococcus* spp. RS3) to control the growth of two strains of* A. parasiticus* and four of* A. flavus* (all aflatoxigenic).* L. plantarum* YO strain significantly inhibited the vegetative growth and sporulation of all phytopathogens tested. These findings demonstrate the antifungal activity of some* Lactobacillus*, and the inhibition was possibly related to a pH reduction and/or a nutrient competition of the culture medium. Bueno et al. [23] evaluated the capacity of two* Lactobacillus* species (*L. casei* CRL 431 and* L. rhamnosus* CRL, 1224) to reduce the growth of three strains of* A. flavus*, and they observed a decreased growth of the phytopathogens when cultivated in association with the* Lactobacillus* strains (reduction of mycelium dry weight of 73% and 85% with CRL 43 and CRL 1224 strain, resp.). Similar results were observed by Muñoz et al. [24], when three LAB and one* S. cerevisiae* strain were tested for antagonistic effects against* A. nomius* under different incubation conditions. After three days of coculture, 75%, 40%, 36%, and 20% of growth inhibition of* A. nomius* were observed for* L. rhamnosus* O236,* L. fermentum* ssp.* cellobiosus* 408,* L. fermentum* 27A, and* S. cerevisiae*, respectively. However, these antagonistic effects against* A. nomius* growth were not accompanied by morphological alterations of the hyphae.

**Figure 2:**
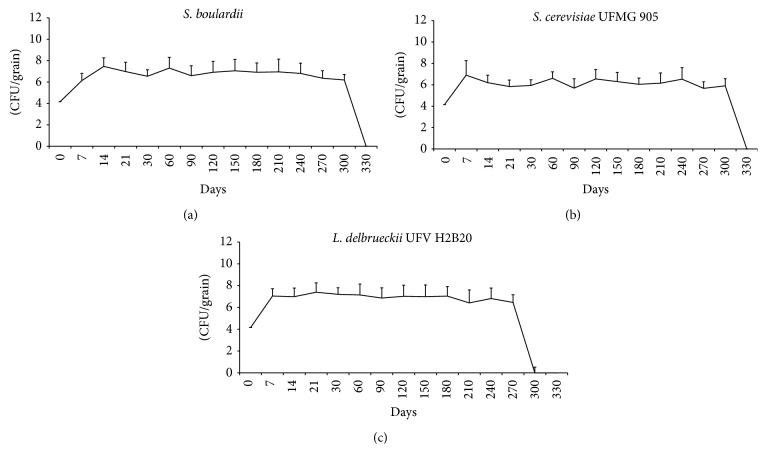
Viable cell count (CFU/Grain) of* S. boulardii* (a),* S. cerevisiae* UFMG 905 (b), and* L. delbrueckii* UFV H2b20 (c) during storage at 30°C for 330 days.

As shown above, better results for biological control of the phytopathogen were obtained when the yeasts and bacteria were used in viable form when compared to the inactivated form. As in Brazil, grains are generally stored during a mean period of about six months, and it was important to evaluate the evolution of viable population levels of the yeasts and bacteria after their inoculation onto the surface of peanuts grains during a simulated storage.  Figure 2 shows that high levels of viable cells were maintained at least until 300 days after sprinkling of the three microorganisms.

Aflatoxin production by* A. parasiticus* was analyzed in the presence of each yeast and bacterium alone or in pair combinations. In the first situation, only the yeasts caused a significant reduction in aflatoxin production (Table 2), with statistically similar effects for* S. cerevisiae* UFMG 905 (72.8%) and* S. boulardii* (65.8%).

When inoculated in pairs, all combinations significantly reduced aflatoxin production by* A. parasiticus* (Table 3). Interestingly, the best result was obtained with the coinoculation of* S. boulardii* and* L. delbrueckii* (96.1%). This synergistic effect between yeast and the* Lactobacillus* was not observed in the combinations between* S. boulardii* and* S. cerevisiae* (71.1%) or* L. delbrueckii* and* S. cerevisiae* UFMG 905 (66.7%). Similar results were obtained by Prado et al. [6] when they used two yeasts pertaining to the* Saccharomycopsis* genus (*S. schoenii* and* S. crataegensis*) as biological control agents. These yeasts were able to reduce* A. parasiticus* production of aflatoxin B_1_ and G_1_ in peanuts. These results suggest that biological control with selected microorganisms could reduce not only the spore dispersion but also the production of mycotoxins by phytopathogens in stored grains.

Reddy et al. [25] used three bacterial species (*Rhodococcus erythropolis*,* Bacillus subtilis and Pseudomonas fluorescens*) for biological control of* A. flavus* and observed that* R. erythropolis* completely inhibited phytopathogen growth and, as a consequence, its aflatoxin B_1_ production. The other microorganisms reduced mycelium growth in a range of 65 to 74% and aflatoxin production from 39% to 65%. The authors also noted that the inhibitory activity is likely due to a chemical antagonistic extracellular substance produced by the bacteria. Prado et al. [19] tested* S. cerevisiae* YEF 186 as an* A. parasiticus* antagonist in two peanut cultivars (IAC Runner and IAC Caiapó) with two different incubation times (seven and fourteen days) and two different inoculation sequences (yeast inoculated simultaneously or three hours before the pathogen). The authors found that the best reduction of aflatoxin B_1_ (74.4%) was obtained after seven days and when the yeast was inoculated before the pathogen. The authors suggested that this reduction was probably due to aflatoxin adhesion to the yeast cell wall or to aflatoxin degradation by the yeast, and this is probably a way of reduction observed in this study. Gerbaldo et al. [26] evaluated the antifungal activities and aflatoxin B_1_ reduction promoted by two* Lactobacillus* species (*L. rhamnosus* L60 and* L. fermentum* L23) with known probiotic activities. The* Lactobacillus* strains were tested against ten aflatoxigenic* Aspergillus* strains (nine* A. flavus* strains and one* A. parasiticus*). They found that* Lactobacillus* L60 and L23 inhibited the mycelia growth of all* Aspergillus* strains tested and promoted a reduction in aflatoxin production from 73 to 99%. They also suggested three possible mechanisms to explain the effect: (1) aflatoxin degradation by enzymes from* Lactobacillus*, (2) competition for space or nutrients, or (3) absorption of aflatoxin onto the cell walls of the* Lactobacillus*.

**Figure 3:**
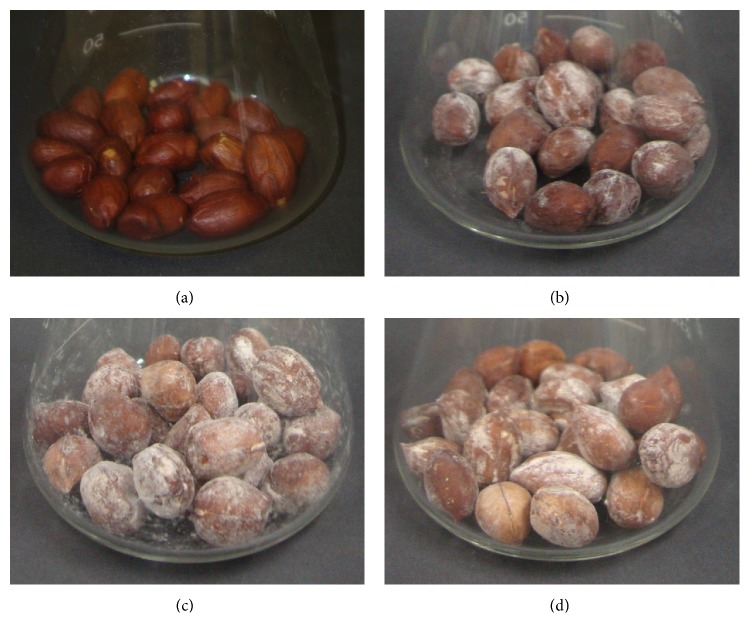
Erlenmeyer flasks with 15 g of autoclaved peanuts cultivar IAC caiapó inoculated with (a) negative control, only 5 mL of 0.1% Tween 80; (b)* S. boulardii* (2.5 mL at 10^8^ cells/mL plus 2.5 mL of 0.1% Tween 80), (c)* S. cerevisiae* UFMG 905 (2.5 mL a 10^8^ cells/mL plus 2.5 mL of 0.1% Tween 80), (d)* L. delbrueckii* UFV H2b20 (2.5 mL at 10^8^ cells/mL plus 2.5 mL of 0.1% Tween 80) incubated at 30°C for 330 days.

Concluding, results of the present study showed that candidate microorganisms for biological control able to reduce the production of spores by aflatoxigenic fungi are more effective in a viable form than in an inactivated form. Additionally, the candidates tested here showed a high capacity to remain viable in high population levels even 300 days after their inoculation on the peanuts grains, which is an important property since in Brazil, the mean storage time for grains is of approximately six months. An interesting characteristic observed was that the tested microorganisms did not produce any apparent harm to the peanuts, and on the contrary, the microbial biofilm gave the grains an appearance similar to salted peanuts (Figures 3(a)–3(d)).

Another advantage of probiotics is that their commercial use in the food industry is not restricted by legislation as is the case with other microorganisms. Finally, the most important effect observed with the sprinkling of the tested microorganisms was the considerable reduction of aflatoxin production by* A. parasiticus*, especially when the combination of* S. boulardii* and* L. delbrueckii* H2b20 was used. Overall, these data suggest that the food industries could use the proposed method as an alternative treatment to control the dispersion and aflatoxin production by phytopathogen, remembering that beyond protection during storage, the method could provide an additional probiotic effect in the digestive tract of consumers after ingestion of the treated grains. However, more studies are needed to clarify the possible impact that lactic acid bacteria and yeasts added to reduce aflatoxins could have on the organoleptic characteristics of the products as well as the exact mechanisms responsible for the reduction of the aflatoxin contents.

## Figures and Tables

**Table 1:** Spores production of *A. parasiticus* IMI 242695 after incubation for seven days at 30°C with *S. boulardii*, *S. cerevisiae* UFMG 905, and *L. delbrueckii* UFV H2b20, in viable or inactivated forms. SR = spore reduction, AP = *A. parasiticus*.

Assays	Live cells		Inactivated cells	
	Average^1^ deviation	SR (%)	Average^1^ deviation	SR (%)
Positive control (AP)	11.9 Aa ± 0.37	—	11.9 Aa ± 0.37	—
*S. boulardii* × AP	6.4 Ba ± 0.36	46,3	9.0 Cb ± 0.42	24,9
*S. cerevisiae* × AP	6.5 Ba ± 0.68	45,8	9.2 Bb ± 0.41	22,7
*L. delbrueckii* × AP	4.9 Ba ± 0.89	59,3	10.2 Bb ± 0.49	14,0

^1^Different letters (uppercased letters for columns and lowercased letters for rows) indicate significant differences according to Tukey's test (*P* < 0.05).

**Table 2:** Mean aflatoxin (*μ*g/kg) production by *A. parasiticus* IMI 242695 incubated for seven days at 30°C in peanut grains cultivar IAC Caiapó and perceptual reduction of aflatoxin induced by *S. boulardii*, *S. cerevisiae* UFMG 905, and *L. delbrueckii* UFV H2b20. AP = *A. parasiticus*.

Assays	Average^1^ S.D.	Reduction (%)
Control (AP)	36,695.7 a ± 14,920.8	—
*S. boulardii* × AP	12,538.9 b ± 9,731.9	65.8
*S. cerevisiae* × AP	9,981.8 b ± 2,244.4	72.8
*L. delbrueckii* × AP	34,265.6 a ± 6,184.2	6.6

^1^Different letters indicate significant differences according to Tukey's test (*P* < 0.05); S.D. = standard deviation.

**Table 3:** Mean aflatoxin (*μ*g/kg) production by *A. parasiticus* IMI 242695 incubated for seven days at 30°C in peanut grains cultivar IAC Caiapó and perceptual reduction of aflatoxin induced by combinations of *S. cerevisiae* UFMG 905, *S. boulardii*, and *L. delbrueckii* UFV H2b20. AP = *A. parasiticus*.

Assays	Average^1^ deviation	Reduction (%)
Control (AP)	15,187.2 a ± 3,957.0	—
*L. delbrueckii* + *S. cerevisiae* × AP	5,062.1 b ± 3,695.0	66.7
*S. boulardii* + *S. cerevisiae* × AP	4,389.4 b ± 334.7	71.1
*S. boulardii* + *L. delbrueckii* × AP	589.4 c ± 516.6	96.1

^1^Different letters indicate significant differences according to Tukey's test (*P* < 0.05).
